# Supplementary material for: Cranial anatomy of Emeroleter levis and the phylogeny of Nycteroleteridae
Source: PLoS One. 2024 Apr 29;19(4):e0298216. doi: 10.1371/journal.pone.0298216 (PMC11057731; doi:10.1371/journal.pone.0298216)
Supplement: Attachment — Submitted filename: Response to Reviewers.docx [file pone.0298216.s002.docx]

**Response to Reviewers**

Kayla D. Bazzana-Adams, Mark J. MacDougall, Valery V. Bulanov, Jörg Fröbisch

We thank the reviewers for their constructive feedback on this manuscript. As follows are point-by-point responses to the comments that were raised by the reviewers.

**Journal Requirements:**

When submitting your revision, we need you to address these additional requirements.

1. Please ensure that your manuscript meets PLOS ONE's style requirements, including those for file naming. The PLOS ONE style templates can be found at 
<https://journals.plos.org/plosone/s/file?id=wjVg/PLOSOne_formatting_sample_main_body.pdf> and 
<https://journals.plos.org/plosone/s/file?id=ba62/PLOSOne_formatting_sample_title_authors_affiliations.pdf>

2. In your manuscript, please provide additional information regarding the specimens used in your study. Ensure that you have reported human remain specimen numbers and complete repository information, including museum name and geographic location.

If permits were required, please ensure that you have provided details for all permits that were obtained, including the full name of the issuing authority, and add the following statement:

'All necessary permits were obtained for the described study, which complied with all relevant regulations.'

If no permits were required, please include the following statement:

'No permits were required for the described study, which complied with all relevant regulations.'

For more information on PLOS ONE's requirements for paleontology and archeology research, see <https://journals.plos.org/plosone/s/submission-guidelines#loc-paleontology-and-archaeology-research>.

- Repository information is already provided in the manuscript under “Institutional Abbreviations”
- Permit statement has been added

3. Thank you for stating the following financial disclosure: 
   "This work was supported by funding from the German Research Foundation (DFG project No. 2457/9-1) and a scholarship to KBA from the Humboldt Internship Program."

Please state what role the funders took in the study.  If the funders had no role, please state: "The funders had no role in study design, data collection and analysis, decision to publish, or preparation of the manuscript." 
If this statement is not correct you must amend it as needed. 
Please include this amended Role of Funder statement in your cover letter; we will change the online submission form on your behalf.

- Role of Funder statement has been added to cover letter

4. We note that you have stated that you will provide repository information for your data at acceptance. Should your manuscript be accepted for publication, we will hold it until you provide the relevant accession numbers or DOIs necessary to access your data. If you wish to make changes to your Data Availability statement, please describe these changes in your cover letter and we will update your Data Availability statement to reflect the information you provide.

**Additional Editor Comments**:

One problem is related to the figures: many discussed features not marked in figures

- Figure 1 take out of mandibles from ventral view
  - Subsequent to our initial submission of this manuscript to PLOS ONE, the institution where this work was carried out was subjected to a cybersecurity breach that has resulted in the loss of access to significant portions of the institutional data repository and to many user’s accounts, including those of the authors. Work is ongoing to restore access, but at present we remain unable to access the segmentation files which are stored in KBA’s institutional account. As a result, we are currently unable to make any changes to figures that would require accessing the segmentation files.
- L170 in my view, only posterior margin
  - Description has been corrected
- L190 groove, mark on figure 2
  - Label has been added to Figure 2
- cp also on figure 2,
  - There is no cultriform process in the specimen described here.
- L240 oval concavity mark it on Fig 3
  - Label has been added to Figure 3
- L274 The surangular is relatively long, extending anteriorly to the level of the anterior end of the coronoid eminence. [No, it is posterior to that]
  - The surangular does extend to the level of the anterior portion of the coronoid eminence; this is visible in both lateral and medial views. We have adjusted the descriptive statement for clarity.
- L280 MARK foramen intermandibularis caudalis on fig4, I cannot see it
  - This identification has been corrected in both the manuscript and figure.
- L295 MATK foramen intermandibularis on Fig 4A
  - This identification has been corrected in both the manuscript and figure.
- orbitonasal ridge and paraseptal cartilage mark on fig2
  - Label has been added for the orbitonasal ridge. We refrain from labelling the paraseptal cartilage, as the cartilage itself is not preserved; our description refers to the fossa defined by the orbitonasal ridge that the cartilage would have occupied.
- meckelian fossa is unclear in your figure 4, mark it
  - Label has been added.

**Comments to the Author**

1. Is the manuscript technically sound, and do the data support the conclusions?

The manuscript must describe a technically sound piece of scientific research with data that supports the conclusions. Experiments must have been conducted rigorously, with appropriate controls, replication, and sample sizes. The conclusions must be drawn appropriately based on the data presented.

Reviewer #1: Partly

Reviewer #2: Yes

2. Has the statistical analysis been performed appropriately and rigorously?

Reviewer #1: I Don't Know

Reviewer #2: Yes

3. Have the authors made all data underlying the findings in their manuscript fully available?

The [PLOS Data policy](http://www.plosone.org/static/policies.action#sharing) requires authors to make all data underlying the findings described in their manuscript fully available without restriction, with rare exception (please refer to the Data Availability Statement in the manuscript PDF file). The data should be provided as part of the manuscript or its supporting information, or deposited to a public repository. For example, in addition to summary statistics, the data points behind means, medians and variance measures should be available. If there are restrictions on publicly sharing data—e.g. participant privacy or use of data from a third party—those must be specified.

Reviewer #1: Yes

Reviewer #2: Yes

4. Is the manuscript presented in an intelligible fashion and written in standard English?

PLOS ONE does not copyedit accepted manuscripts, so the language in submitted articles must be clear, correct, and unambiguous. Any typographical or grammatical errors should be corrected at revision, so please note any specific errors here.

Reviewer #1: Yes

Reviewer #2: Yes

**Review Comments to the Author**

**Reviewer #1:**

The paper “Cranial anatomy of Emeroleter levis and the phylogeny of the Nycteroleteridae” is an interesting paper with new and important morphological data from a poorly-known yet not-insignificant clade. The methodology for the CT scanning and segmentation seems to be sound and the resulting figures and description are valuable.

Only a couple of typos of note: Line 110: “boarder” should be “border”; Line 446-7: “examination of the taxon” should be “examination of additional taxa” or the like.

- Line 110: “Boarder” has been corrected to “border”
- Line 446-7: “examination of the taxon” is correct, as we are referring to examining *Lanthanosuchus* with computed tomography.

The anatomical description is complete; it may be a personal preference for a single comparative description within the results, rather than having the comparisons contained separately within the discussion. However, if the comparison is to feature in the discussion, I think it would add to the relevance of the paper if this was done within a phylogenetic framework. Is any of this morphology relevant to the phylogenetic analysis completed in the paper?

- We prefer to keep the description and comparison sections separate, and to provide comparisons by element rather than by phylogenetic grouping, to facilitate ease of reference by future workers.
- Various aspects of the new and/or revised morphology contribute to the revised phylogenetic analysis and these are discussed in the relevant sections. Although many of the anatomical features discussed in the comparative section do not directly impact the phylogenetic analysis, we believe the value of comparative information warrants inclusion of these comparisons in the discussion section.

However; there are major issues in the phylogenetic analysis and systematic palaeontology that should be addressed before acceptance and publication.

Phylogenetic analysis:

- Usually at least 10 000 replicates are done in the heuristic search. More replicates would not greatly increase the analysis time, but would result in a better analysis. Also a Bayesian analysis would not be out of place here if you are comparing results to papers that use the method.
  - Replicates were increased to 10 000. We prioritize the use of a parsimony analysis for comparability with previous studies of nycteroleterids, as it is historically parsimony analyses that have failed to produce a resolved Nycteroleteridae.
- Also, some of the characters that have been re-scored for Emeroleter are based off this single specimen, which is likely juvenile and also likely crushed dorso-ventrally: ie. rescoring “snout shape wider than tall” when the character scoring was based on other specimens may not be valid.
  - In the original paper describing *Emeroleter*, the snout also appears wider than tall and is reconstructed as such. This is also the condition seen in other nycteroleters, so we believe this coding to be appropriate based on current knowledge of the taxon and of nycteroleters in general. However, retrodeformation of the specimen in the future, once access to the segmentation files is restored, would be interesting.
- Also, some traits seem to be variable, such as “jugal anterior process extends to al least the level of the anterior orbital rim”, so should be scored as variable.
  - Traits have been re-scored where appropriate.

Revised diagnosis:

- The characters that are named as autapomorphies of Emeroleter are not, in fact, autapomorphies of the taxon. For example, “maxilla extends to and contributes to the posterodorsal margin of the naris” is common to all ‘nycteroleters’. Please re-evaluate all characters in this section.
  - Characters in the revised diagnosis have been updated, with the removal of more equivocal characters and addition of characters not in the matrix. However, we do not agree that the contribution of the maxilla to the posterodorsal margin of the naris is in fact common to all nycteroleters, so we have left this character in the diagnosis.

Referred specimen: mention that the specimen is likely juvenile and has experienced dorso-ventral crushing.

- Comment has been added.

Phylogenetic Analysis:

- Please show the results of the strict consensus in the analysis. There are a number of papers (ie. Sharkey and Leathers 2001 in Cladistics, others) that show majority rules consensus is “inappropriate” – just because one topology occurs more often doesn’t mean it is “more correct”. Plus you cannot compare the 50% majority-rules consensus tree to the strict consensus trees of other papers. This analysis had 53 MPTs, so a strict consensus tree would have unresolved branches seen in other analyses. This also means that the “Phylogenetic implications” section needs to be re-written with this in mind. There are only fully-resolved clades because of the use of a consensus tree where other papers did not display the data this way.
  - Results have been updated to include the strict consensus tree, which has an identical topology for Nycteroleteridae.

General comments:

- Is there anything in the new data from the braincase anatomy that supports or refutes the hypothesis that nycteroleters had particularly good hearing (Mueller and Tsuji 2007)? I also think the final sentence need to be re-written/be stronger.
  - Discussion of the auditory morphology in *Emeroleter* and comparison to *Macroleter* has been added to final section of discussion; as a result, the final sentence has also been changed.

Figures: Figures are good, but it is difficult to perceive depth particularly in the dorsal and - ventral views (Figure 1 A and B); is there a setting on Amira that can ‘add depth’ to the image?

- See response to editor’s comments above, regarding loss of access to segmentation files.

Figure 5: the ‘Bashkyroleters’ should be in single quotes as even here they aren’t monophyletic.

- Quotes have been added to Figure 5.

**Reviewer #2:**

This work provides detailed anatomical information on a Permian reptile from Russia. One of the main results is an improved phylogenetic placement based on the most recent version of the data matrix. The work is well written, the results are solid and the figures are of great quality. I have no other comments to make, except two minor ones:

I replicated the phylogenetic analysis on TNT with the data base and the parameters provided in the text. The program found 53 trees as well, but reported a shorter length (783) which differs from the length reported here (787). The topology of the consensus tree, however, is identical. Please double check the tree length.

- Tree length has been updated, as we have revised the matrix slightly based on the comments of Reviewer 1. Tree length was found to be 788 using PAUP.

The taxon "*Owenetta kitchingorum*" mentioned in the analysis is now considered a junior synonym of *Saurodektes kitchingorum* (Hamley et al. 2020: <https://doi.org/10.1093/zoolinnean/zlaa056>). *Owenetta* is also mentioned several times in comparisons within the text. The authors should check if they are talking about the Permian *Owenetta rubidgei* or the Triassic *Saurodektes kitchingorum* (= *Owenetta kitchingorum*).

- References to ‘*Owenetta*’ have been updated to *Saurodektes*.

Great work!

6. PLOS authors have the option to publish the peer review history of their article ([what does this mean?](https://journals.plos.org/plosone/s/editorial-and-peer-review-process#loc-peer-review-history)). If published, this will include your full peer review and any attached files.

If you choose “no”, your identity will remain anonymous but your review may still be made public.

**Do you want your identity to be public for this peer review?** For information about this choice, including consent withdrawal, please see our [Privacy Policy](https://www.plos.org/privacy-policy).

Reviewer #1: No

Reviewer #2: **Yes:** Juan Carlos Cisneros
